# Immobilization of Perylenetetracarboxylic Dianhydride on Al_2_O_3_ for Efficiently Photocatalytic Sulfide Oxidation

**DOI:** 10.3390/molecules29091934

**Published:** 2024-04-24

**Authors:** Jiahao Liang, Jie Wang, Hao Hou, Qingzhu Xu, Wei Liu, Chenliang Su, Hongli Sun

**Affiliations:** 1 International Collaborative Laboratory of 2D Materials for Optoelectronic Science & Technology, Engineering Technology Research Center for 2D Materials Information Functional Devices and Systems of Guangdong Province, Institute of Microscale Optoelectronics, Shenzhen University, Shenzhen 518060, China; 18844536117@163.com (J.L.); houhao@szu.edu.cn (H.H.); mz120210747@yzu.edu.cn (Q.X.); lw0584@szu.edu.cn (W.L.); chmsuc@szu.edu.cn (C.S.); 2 State Key Laboratory of Radio Frequency Heterogeneous Integration, Shenzhen University, Shenzhen 518060, China

**Keywords:** heterogeneous organic photocatalysts, photoredox, O_2_ reduction, strong adsorption of thioanisole, thioanisole oxidation

## Abstract

Perylenetetracarboxylic dianhydride (PTCDA) derivatives have received significant attention as molecule photocatalysts. However, the poor recyclability of molecule-type photocatalysts hinders their widespread applications. Herein, immobilization of PTCDA on Al_2_O_3_ was achieved by simply physical mixing, which not only dramatically improved their recyclability, but also surprisingly improved the reactivity. A mechanism study suggested that the photo-exited state (PTCDA*) of PTCDA could promote the oxidation of thioanisole to generate PTCDA^•−^, which sequentially reduces oxygen to furnish superoxide radicals to achieve the catalytic cycle. Herein, the immobilization support Al_2_O_3_ was able to facilitate the strong adsorption of thioanisole, thereby boosting the photocatalytic activity. This work provides a new insight that the immobilization of organic molecular photocatalysts on those supports with proper adsorption sites could furnish highly efficient, stable, and recyclable molecular-based heterogeneous photocatalysts.

## 1. Introduction

Photoredox catalytic organic reactions have attracted increasing attention [1]. Photoredox catalysts, mainly including metal complexes, dye molecules, and polymers, have been widely applied in many classic redox reactions (dehydrogenation, carbonyl reduction, and alcohol oxidation etc.) [2]. 3,4,9,10-Perylenetetracarboxylic dianhydride (PTCDA), as one of the molecule-type photoredox catalysts, features a broad light absorption range and suitable redox property, which can achieve dehalogenation reactions, aerobic oxidation reactions, etc. [1,3]. A typical example is that PTCDA molecules can simultaneously achieve the oxidation of alcohols and the reduction of protons to release H_2_ [3]. In addition, the imines derived from PTCDA can also serve as photocatalysts for dye degradation, wastewater treatment, etc. [4,5,6,7,8]. However, since PTCDA is a molecule photocatalyst, it is inevitable that issues such as photo-stability and difficulty will be encountered in catalyst separation and recovery. Therefore, improving its stability and recyclability by heterogenization, and still retaining and even improving its redox capabilities, is highly attractive. There are three main categories of heterogenization methods: the first one is to construct polymer/supermolecules semiconductors via pyrolysis etc. using PTCDA and its derivatives as precursors [9,10]; the second is to combine PTCDA-converted polymers/supermolecules with oxides [11,12,13,14]; and the third is to act as the functional monomers to modify the other polymers [15,16]. In the second strategy, the interaction between the PTCDA-based materials and the oxide is potentially highly important, but rarely explored. Thus, herein, we wish to immobilize PTCDA on oxides (e.g., Al_2_O_3_) and probe the catalytic effects of their interaction. Specifically, a simple grinding method was used to physically mix PTCDA with metal oxides, where Al_2_O_3_ was screened out as a proper support for PTCDA in Figure S1. As revealed by the XPS analysis and theoretical studies, this simple grinding procedure could induce strong interaction between the anhydride groups of PTCDA and Al_2_O_3_. FTIR analysis reflected that Al_2_O_3_ with Lewis acidity could provide sufficient molecular adsorption sites for reagents. Benefiting from the synergistic effects between PTCDA and Al_2_O_3_, the photocatalytic activity of PTCDA/Al_2_O_3_ was significantly higher than that of PTCDA. A mechanism study suggested that this immobilization strategy helps to enhance the absorption of reagents such as thioanisole, accelerate its conversion to the corresponding radicals, and thus promote the subsequent oxidation by the superoxide radicals from oxygen reduction.

## 2. Results and Discussion

### 2.1. Synthesis, Activity Evaluation, and Interaction Study of PTCDA/Al_2_O_3_ Mixture

**Figure 1:**
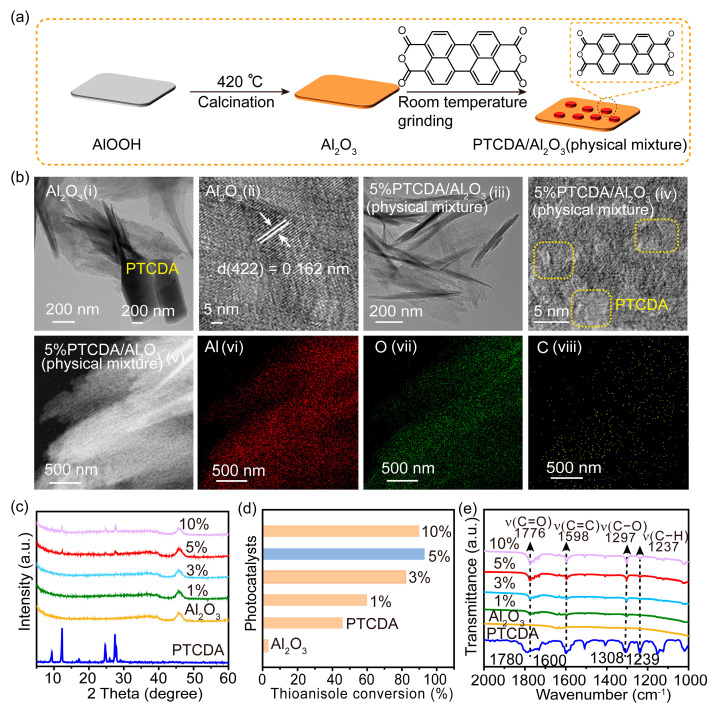
(**a**) The preparation scheme of PTCDA/Al_2_O_3_(physical mixture). (**b**) TEM (**i**,**iii**), and HRTEM (**ii**,**iv**) images of Al_2_O_3_ and PTCDA/Al_2_O_3_ (physical mixture); the insert is the image of original PTCDA. EDS mapping images (**v**–**viii**) of PTCDA/Al_2_O_3_ (physical mixture). (**c**) XRD patterns, (**d**) The photocatalytic activities and (**e**) FTIR spectra of PTCDA, Al_2_O_3_, and PTCDA/Al_2_O_3_ (physical mixture) with different mass ratios of PTCDA. Reaction conditions: photocatalyst, 10 mg; methanol, 2 mL; 0.1 mmol, thioanisole; white LED (≥420 nm), 500 mW cm^−2^; O_2_, 1 atm; time, 5 h.

Al_2_O_3_, as an insulator with surface hydroxyl and Lewis acid sites [17,18], was screened out as a superior support for PTCDA (Figure S1), as it can provide synergistic sites for both O_2_ and substrates. The preparation of PTCDA/Al_2_O_3_(physical mixture) is shown in Figure 1a. Through the TEM images of Al_2_O_3_ and PTCDA in Figure 1b, Al_2_O_3_ exhibited a layer morphology, while PTCDA had a rod-shaped morphology. And a crystal plane spacing of 0.162 nm was displayed, belonging to the (422) plane of Al_2_O_3_. After the physical mixture of Al_2_O_3_ and PTCDA was grounded, the rod-shaped PTCDA disappeared and was replaced by a morphology similar to Al_2_O_3_. It could be observed from HRTEM images (Figure 1b, iv) that there were certain amorphous substances on the surface of Al_2_O_3_, which could be classified as PTCDA molecules. EDS mapping also demonstrated the uniform distribution of PTCDA on the surface of Al_2_O_3_. The XRD patterns in Figure 1c confirmed that the diffraction peaks of PTCDA molecules gradually increased as the amount of PTCDA molecules increased, demonstrating the successful incorporation of PTCDA molecules into Al_2_O_3_. UV-Vis spectra reflected the light absorption properties of PTCDA and Al_2_O_3_ (Figure S2). Compared with Al_2_O_3_, PTCDA has significant absorption within the visible light absorption range, implying that this heterogenization strategy has the potential to improve the photocatalytic activity of PTCDA/Al_2_O_3_ (physical mixture). According to the reported literature [3], the reduction potential (−0.27 V vs. NHE, pH = 0) of PTCDA is more negativethan that (−0.13 V vs. NHE, pH = 0) of O_2_/•O_2_^−^, suggesting the possibility and feasibility of activating oxygen after mixing PTCDA and Al_2_O_3_. Subsequently, the photocatalytic activities toward selective oxidation of sulfide over the series of catalysts were evaluated (Figure 1d). Compared to independent Al_2_O_3_ or PTCDA, the heterogenized PTCDA showed a significant improvement in photocatalytic activity, where 5%PTCDA/Al_2_O_3_ gave the best performance.

**Figure 2:**
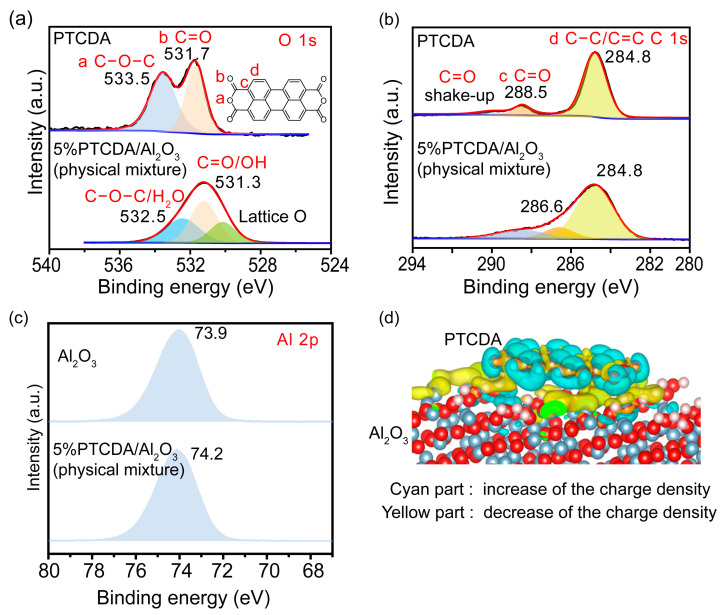
High-resolution XPS spectra of (**a**) O 1s, (**b**) C 1s, and (**c**) Al 2p for 5%PTCDA/Al_2_O_3_ (physical mixture) and PTCDA or Al_2_O_3_. (**d**) The calculated differential charges of PTCDA on Al_2_O_3_ surface.

To understand the reasons behind the unexpectedly boosted performance of the heterogenized PTCDA, the interaction between the PTCDA and Al_2_O_3_ was further investigated by FTIR (Figure 1d). Compared with pure PTCDA, the bands of C=O and C−O of heterogenized PTCDA showed significant red shifts, while there were no significant shifts in C−H or C=C, confirming that the interaction was likely to occur between the anhydride group of PTCDA and Al_2_O_3_. In great contrast to the minor shift of hydroxyl groups in calcinated Al_2_O_3_, the significant blue shift of hydroxyl groups induced by the calcination of the PTCDA/Al_2_O_3_ (physical mixture) and the apparent red shift in the characteristic infrared peaks of C=O and C−O, further confirmed that the anhydride group of PTCDA mainly interacted with the hydroxyl groups on the surface of Al_2_O_3_ (Figure S3). The interaction between the two was also studied by XPS (Figure 2a–c). Differently from the basically unchanged binding energy of C 1s in C−C or C=C, the binding energies of O 1s and C 1s in C−O−C and C=O decreased, and the binding energy of Al 2p increased, confirming that the mutual interaction would increase the electron density of PTCDA molecules and decrease the electron density on the surface of Al_2_O_3_ due to the electron-pulling effect of the anhydride group after the interaction. The calculated differential charge density (yellow) represents an increase in charge density, while cyan represents a decrease in charge density in Figure 2d. It was also demonstrated that the adsorption on the Al_2_O_3_ surface would increase the electron density of PTCDA molecules, as is consistent with the results of XPS.

### 2.2. Exploration of the Mechanism

**Figure 3:**
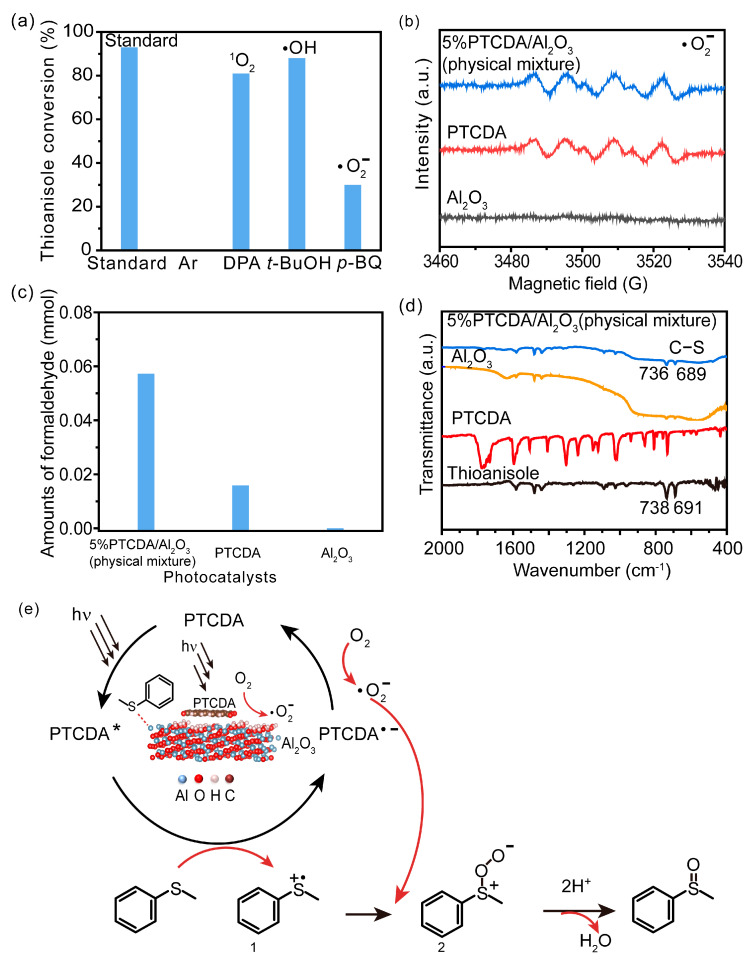
(**a**) The influence of different sacrificial agents on photocatalytic performances. *t*-butyl alcohol (*t*-BuOH), nitrotetrazolium blue chloride (NBT), and 9,10-diphenylanthrene (DPA) were used as the sacrificial agents to probe the roles of hydroxyl radical (•OH), superoxide radical (•O_2_^−^), and singlet oxygen (^1^O_2_), respectively [19]. (**b**) EPR signals of the photocatalytically generated DMPO-•O_2_^−^ over Al_2_O_3_, PTCDA, and 5%PTCDA/Al_2_O_3_(physical mixture). (**c**) The effects of different catalysts on formaldehyde production. Reaction conditions: photocatalyst, 10 mg; methanol, 2 mL; white LED (≥420 nm), 500 mW cm^−2^; O_2_, 1 atm. (**d**) FTIR spectra of Al_2_O_3_, PTCDA, and 5%PTCDA/Al_2_O_3_ (physical mixture) after adsorbing thioanisole. (**e**) The proposed mechanism of thioanisole oxidation over 5%PTCDA/Al_2_O_3_ (physical mixture).

Scavenger studies were conducted to determine the effects of various reactive species (Figure 3a). The phenomenon of almost no activity under an argon atmosphere proved the importance of oxygen. Furthermore, the quenching of ^1^O_2_ and •OH did not induce an obvious reduction in activity, indicating that these two oxygen reactive species were not involved in the oxidation reaction. Instead, the addition of •O_2_^−^ sacrificial agents significantly reduced the reaction activity, confirming that •O_2_^−^ was the main active species in the reaction system. Even though the •O_2_^−^ radicals were important in the reaction system, the intensity of •O_2_^−^ signals of PTCDA and heterogenized PTCDA under visible light irradiation (Figure 3b) were similar, indicating that the enhanced performances probably did not originate only from oxygen-reduction parts. We thus started to probe the influence of substrate oxidation using methanol as a probing reagent, and the Nash detection method was used to detect the presence of formaldehyde. It was found that the formaldehyde generated by the PTCDA/Al_2_O_3_ (physical mixture) system was 6 times higher than that of the PTCDA system, confirming that the oxidation parts were also accelerated. To further understand the remarkable enhancement, the methanol adsorption experiments were studied by FTIR (Figure S4). Classical and weak absorption peak of −CH_x_ of methanol was observed over Al_2_O_3_ and 5%PTCDA/Al_2_O_3_ (physical mixture), but not over PTCDA, indicating the preferential adsorption of methanol on the surface of Al_2_O_3_ compared to PTCDA. Similarly, the enhanced adsorption of thioanisole on 5%PTCDA/Al_2_O_3_ (physical mixture) was also observed (Figure 3d). Thus, it can be deduced that in the heterogenized mixture, Al_2_O_3_ would favor the adsorption of substrate, and PTCDA could activate O_2_ to furnish •O_2_^−^ radicals, then work synergistically to boost the oxidation activity of sulfide. Therefore, a reasonable mechanism was proposed (Figure 3e). Under visible light irradiation, PTCDA could be excited to the excited state, PTCDA*, which had the potential to oxidize the surface-adsorbed sulfide to corresponding intermediate radical 1, and itself was converted to PTCDA^•−^. Then, O_2_ could obtain electrons from PTCDA^•−^ to produce •O_2_^−^ and further oxidize intermediate 1 to intermediate 2, then follow proton abstraction to produce sulfoxide. Table 1 shows the reaction scope. The substrate-bearing electron donating or weak electron-withdrawing groups all gave high conversion rates and yields for production of the corresponding sulfoxides. And 1 g of thioanisole was used as a substrate to conduct the large-scale photocatalytic reaction under visible light irradiation. The conversion (92%) of thioanisole and yield (62%) of benzyl sulfoxide are given in Table S2, indicating that substrate could be scaled up to the gram level. There was no significant change in photocatalytic activity after multiple cycles (Figure S5a), and FIIR spectra (Figure S5b) confirmed its stability. Overall, the heterogenized PTCDA photocatalysts exhibited good universality and excellent recyclability.

## 3. Materials and Methods

### 3.1. Reagents and Solvents

Aluminum nitrate nonahydrate (Al(NO_3_)_3_•9H_2_O), urea, 3,4,9,10-perylene tetracarboxylic dianhydride (PTCDA, 98%), anisole, methanol, and thioanisole were all purchased from Energy Chemical and used without further purification.

### 3.2. Preparation of 2D Al_2_O_3_ Nanosheets as Precursor

Two-dimensional Al_2_O_3_ nanosheets were prepared using a hydrothermal method with Al(NO_3_)_3_•9H_2_O as raw material [18,20]. A total of 4.5 g of Al(NO_3_)_3_•9H_2_O and 5.1 g of urea were added to a 250 mL round bottom flask, and then 250 mL of ultrapure water was added and maintained at 150 °C for 24 h to obtain a white precipitate. After cooling, the obtained white precipitate was washed with ultrapure water to pH = 7.0 and then dried in an oven at 75 °C for 24 h to obtain the AlOOH precursor. The obtained AlOOH obtained was further calcined in an air atmosphere at 420 °C for 8 h to obtain Al_2_O_3_ nanosheets.

### 3.3. Preparation of PTCDA/Al_2_O_3_(Physical Mixture)

Typically, the synthesis process of 5%PTCDA/Al_2_O_3_ is as follows: 0.0105 g of PTCDA is mixed with 0.2 g of Al_2_O_3_, thoroughly grinding in a mortar until the material is evenly mixed, resulting in a composite material of PTCDA and Al_2_O_3_, labeled as 5%PTCDA/Al_2_O_3_ (physical mixture). A series of x%PTCDA/Al_2_O_3_ was also fabricated by changing the mass percentages of PTCDA, where x denotes 1, 3, 5, and 10, respectively.

### 3.4. General Procedure for the Photocatalytic Selective Oxidation of Sulfide

As is typical, 10 mg of photocatalyst, 1 mL of methanol, and 0.1 mmol of substrate were added to a 10 mL of Pyrex reactor. Then, the reactor was purged with O_2_ for 1 min and maintained at 1 atmosphere. And the reactor was placed in an oil bath at 30 °C for 6 h. Afterwards, 0.01 mmol of 1,3,5-trimethoxybenzene was added as the internal standard, and then the photocatalyst was filtered. The conversion and yield of benzyl sulfoxide were determined using Agilent Technologies 7820 gas chromatography equipped with a WondaCap 5 column with anisole as the internal standard. The conversion and yield of other products ere determined by ^1^H NMR spectra, with 1,3,5-trimethylbenzene as internal standard.

### 3.5. Characterizations

The XRD patterns were obtained using a Rigaku Ultima IV X-RAY diffractometer with Cu Kα radiation (λ = 1.5418 Å) in the 2*θ* range from 5 to 80° at a scanning rate of 8° min^−1^. Transmission electron microscopy (TEM) measurements were carried out using a JEOL F200 microscope (JEOL, Tokyo, Japan) with an accelerating voltage of 200 kV. X-ray photoelectron spectroscopy (XPS) was conducted using a Escalab 250Xi spectrometer at (Thermo, Waltham, MA, USA) room temperature with an Al Kα X-ray source (*hν* = 1486.6 eV). The C 1s peak at 284.8 eV was used as the reference for the calibration of the binding energy. UV-vis diffuse reflectance spectra were measured on an Agilent Technologies Cary Series UV-vis-NIR spectrometer (Agilent, Santa Clara, CA, USA). Fourier Transform infrared spectroscopy (FTIR) measurements were made using a Bruker VERTEX 70v spectrometer (Bruker, Billerica, MA, USA). Diffuse reflectance infrared Fourier transform spectroscopy (DRIFTs) measurements were made using a Bruker Tensor II spectrometers (Bruker, Billerica, MA, USA).

### 3.6. Computational Details

All calculations were performed in the framework of the density functional theory (DFT), with the projector-augmented plane–wave method, as implemented in the Vienna ab initio simulation package (VASP) [21]. The (111) surface was chosen as the active surface to represent the as-prepared γ-Al_2_O_3_ in our calculation model. The vdW interactions were included using the DFT-D2 method of Grimme [22]. The cut-off energy was set to 400 eV. The energy criterion was set to 10^−5^ eV in the iterative solution of the Kohn–Sham equation. The Brillouin zone integration was performed using a 2 × 2 × 1 k-mesh. All the structures were relaxed until the residual forces on the atoms had declined to less than 0.02 eV/Å.

## 4. Conclusions

In summary, a quite simple immobilization strategy was applied to modify the PTCDA molecules, which furnished heterogenized PTCDA with remarkably enhanced reactivity and excellent recyclability. Our studies revealed that the simple grinding procedure could induce a strong interaction between the anhydride group of PTCDA and the hydroxyl group of Al_2_O_3_. Al_2_O_3_ with Lewis acidity provided anchoring sites for reagents to significantly facilitate the electron transfer from reagent to PTCDA*, thus greatly improving the overall performance. This work provides new insights for the low-cost production of superior heterogenized molecular photocatalysts, which lays a foundation for large-scale photosynthesis of fine chemicals and pharmaceuticals using molecular photocatalysts.

## Figures and Tables

**Table 1:**
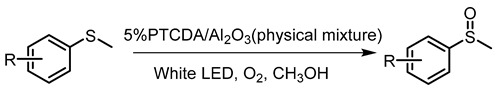
Universality study over 5%PTCDA/Al_2_O_3_ (physical mixture).

Entry	Substrates	Products	Time (h)	Conv. (%)	Sulfoxide Yield(%)
1	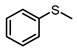	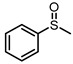	6	93	68
2	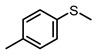	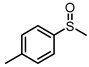	6	94	69
3	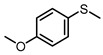	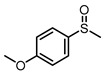	6	99	94
4	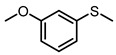	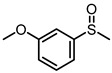	6	91	72
5	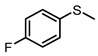	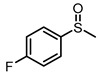	6	94	79
6	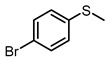	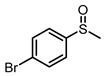	6	96	79
7	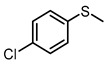	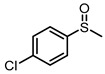	8	91	47
8	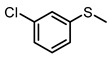	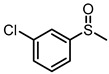	8	96	64

**Reaction conditions**: Photocatalyst, 10 mg; methanol, 2 mL; substrate, 0.1 mmol; white LED (≥420 nm), 500 mW cm^−2^; O_2_, 1 atm. The conversion and yield of benzyl sulfoxide was determined by GC-MS with anisole as internal standard. The conversion and yield of other products was determined by ^1^H NMR spectra with 1,3,5-trimethylbenzene as internal standard.

## Data Availability

Data are contained within the article or Supplementary Materials.

## Supplementary Materials

The following supporting information can be downloaded at: https://www.mdpi.com/article/10.3390/molecules29091934/s1, Figure S1. Comparison of activity of series photocatalysts; Figure S2. UV-vis absorption spectra of PTCDA and Al_2_O_3_; Figure S3. (a) DRIFTs spectra of Al_2_O_3_, PTCDA/Al_2_O_3_(physical mixture), and PTCDA/Al_2_O_3_(physical mixture)−300, (b) DRIFTs spectra of PTCDA and PTCDA calcined at 300 °C (PTCDA−300), (c) DRIFTs spectra of PTCDA and PTCDA calcined at 300 °C (PTCDA−300); Figure S4. FTIR spectra of Al_2_O_3_, PTCDA, and 5%PTCDA/Al_2_O_3_(physical mixture) adsorbing methanol; Figure S5. (a) Evaluation of photocatalytic stability, (b) FTIR spectra of 5%PTCDA/Al_2_O_3_(physical mixture) before and after reaction; Table S1. Control experiments to study photocatalytic sulfide oxidation; Table S2. Control experiments to study photocatalytic sulfide oxidation.
