# Supplementary material for: Immobilization of Perylenetetracarboxylic Dianhydride on Al2O3 for Efficiently Photocatalytic Sulfide Oxidation
Source: Molecules. 2024 Apr 24;29(9):1934. doi: 10.3390/molecules29091934 (PMC11085609; doi:10.3390/molecules29091934)
Supplement: Supplementary file 1 [file molecules-29-01934-s001.zip › molecules-2970575-supplementary.pdf]

Immobilization of Perylenetetracarboxylic Dianhydride on Al_2O_3 for Efficiently Photocatalytic Sulfide Oxidation

Jiahao Liang [1,2], Jie Wang [1,2,*], Hao Hou [1,2], Qingzhu Xu [1,2], Wei Liu [1,2], Chenliang Su [1,2], and Hongli Sun [1,2,*]

[1]International Collaborative Laboratory of 2D Materials for Optoelectronic Science & Technology, Engineering Technology Research Center for 2D Materials Information Functional Devices and Systems of Guangdong Province, Institute of Microscale Optoelectronics, Shenzhen University, Shenzhen 518060, China

[2]State Key Laboratory of Radio Frequency Heterogeneous Integration, Shenzhen University, Shenzhen 518060, China

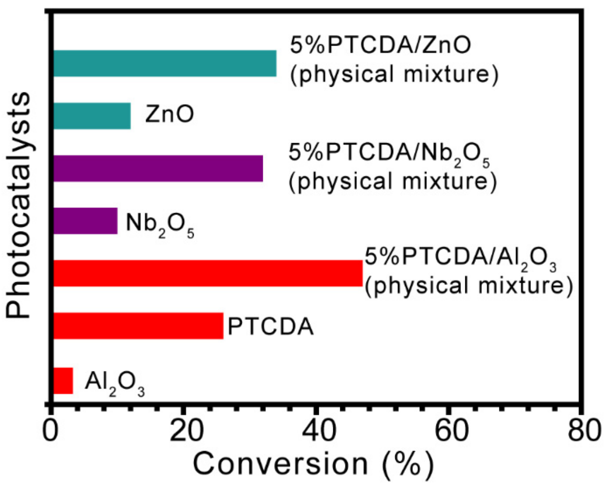

Figure S1 Comparison of activity of series photocatalysts. Reaction conditions: photocatalyst, 10 mg; methanol, 2 mL; substrate, 0.1 mmol; white LED (≥420 nm), 500 mW cm^{-2}; O_2, 1 atm.

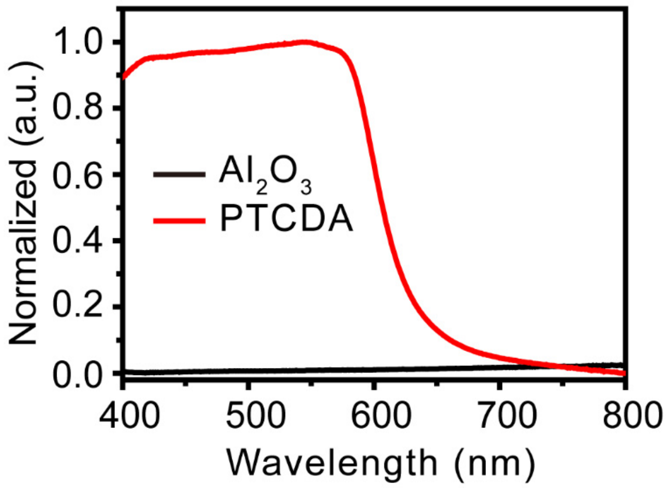

Figure S2 UV-Vis absorption spectra of PTCDA and Al_2O_3.

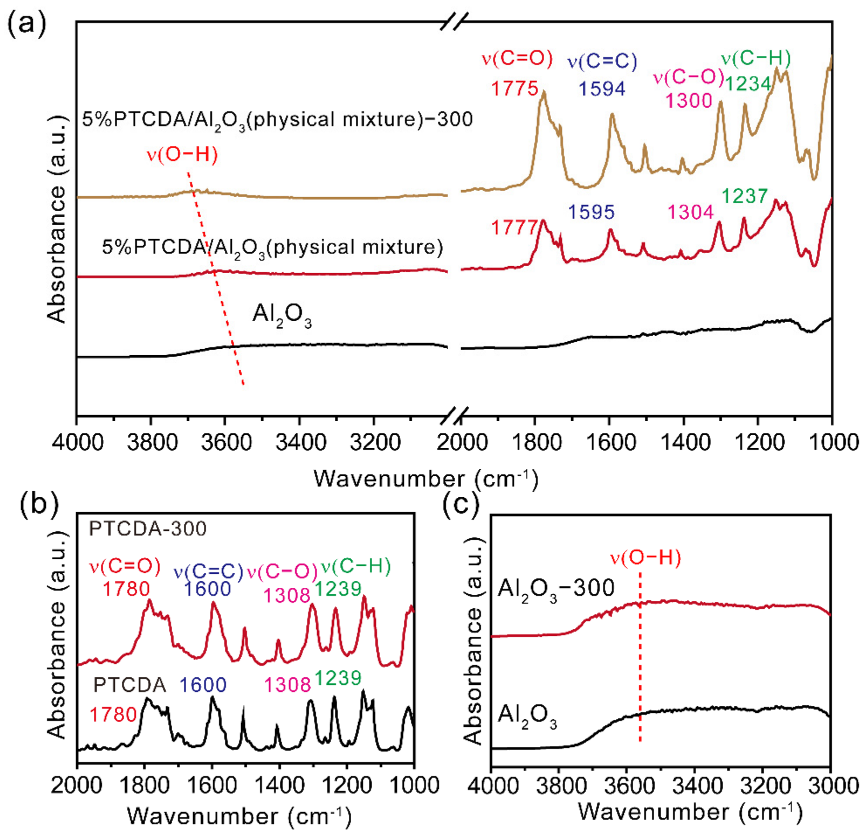

Figure S3 (a) DRIFTs spectra of Al_2O_3, PTCDA/Al_2O_3(physical mixture), and PTCDA/Al_2O_3(physical mixture)−300. PTCDA/Al_2O_3(physical mixture) calcining at 300 °C is symboled as PTCDA/Al_2O_3(physical mixture)−300. (b) DRIFTs spectra of PTCDA and PTCDA calcined at 300 °C (PTCDA−300). (c) DRIFTs spectra of PTCDA and PTCDA calcined at 300 °C (PTCDA-300).

Table S1. Control experiments to study photocatalytic sulfide oxidation.

Entry	Photocatalysts	Time (h)	Atmosphere	Conversion (%)
1	5%PTCDA/Al_2O_3(physical mixture)	6	Ar	0
2	5%PTCDA/Al_2O_3(physical mixture)	6	O_2	93
3	No	6	O_2	0
[a]4	5%PTCDA/Al_2O_3(physical mixture)	6	O_2	0

Reaction condition: photocatalyst, 10 mg; methanol, 2 mL; 0.1 mmol, thioanisole; white LED (≥420 nm), 500 mW cm^{-2}; O_2, 1 atm; time, 6 h. [a]The reaction is conducted in dark.

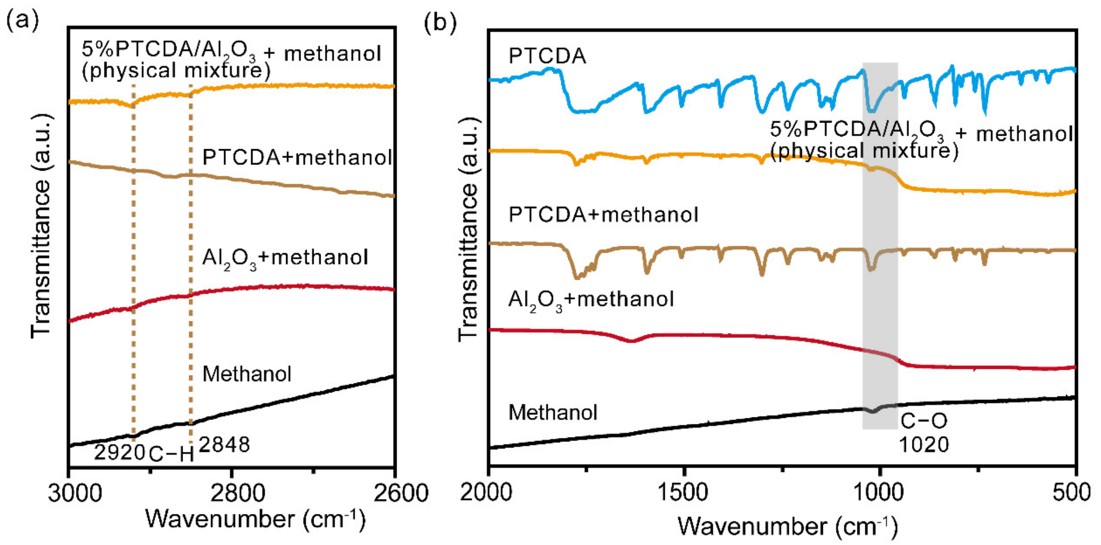

Figure S4 FTIR spectra of Al_2O_3, PTCDA, and 5%PTCDA/Al_2O_3(physical mixture) adsorbing methanol.

Table S2. Control experiments to study photocatalytic sulfide oxidation.

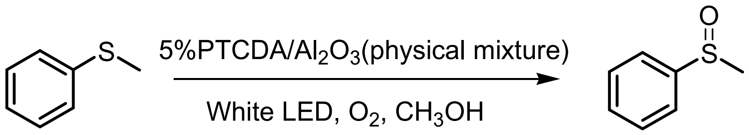

Entry	Photocatalysts	Time (h)	Conversion (%)	Yield (%)
1	5%PTCDA/Al_2O_3(physical mixture)	9	92	62

Reaction condition: photocatalyst, 50 mg; methanol, 25 mL; 1 g, thioanisole; white LED (≥420 nm), 500 mW cm^{-2}; O_2, 1 atm.

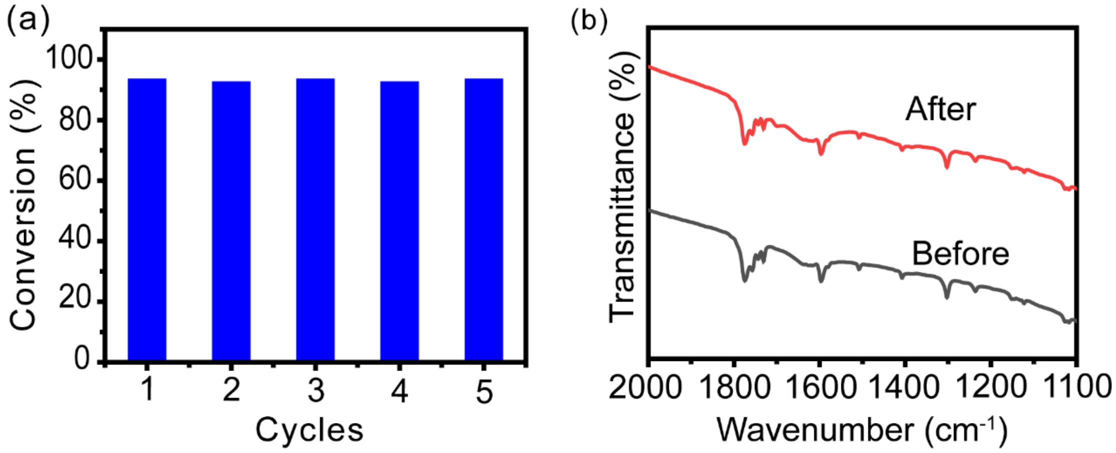

Figure S5 (a) Evaluation of photocatalytic stability. (b) FTIR spectra of 5%PTCDA/Al_2O_3(physical mixture) before and after reaction.
